# Detection of renal brush border membrane enzymes for evaluation of renal injury in neonatal scleredema

**DOI:** 10.12669/pjms.311.5740

**Published:** 2015

**Authors:** Qing Ren, Yongjun Zhang, Jinying Yang, Lixia Wei, Lili Zhao, Qiaozhi Yang

**Affiliations:** 1 Qing Ren, MD, Department of Pediatrics, Liaocheng People’s Hospital, Liaocheng, Shandong252000, China.; 2 Yongjun Zhang, MD, Department of Pediatrics, Liaocheng People’s Hospital, Liaocheng, Shandong252000, China.; 3 Jinying Yang, MD, Center for Disease Control and Prevention of Liaocheng, Liaocheng, Shandong 252000, China.; 4 Lixia Wei, MD, Department of Pediatrics, Liaocheng People’s Hospital, Liaocheng, Shandong252000, China.; 5 Lili Zhao, MD, Department of Pediatrics, Liaocheng People’s Hospital, Liaocheng, Shandong252000, China.; 6 Qiaozhi Yang, MD, Department of Pediatrics, Liaocheng People’s Hospital, Liaocheng, Shandong252000, China.

**Keywords:** β2-microglobulin, Ligustrazine, Neonatal scleredema, Renal brush border membrane enzyme, Renal injury

## Abstract

**Objective:**

To evaluate renal brush border membrane enzymes in urine as an indicator for renal injury in neonatal scleredema (NS).

**Methods:**

Sixty nine NS patients in our hospital were enrolled and divided into mild group and moderate/severe group. Patients were further randomly divided into therapy and control subgroups for 7 days ligustrazine administration. Urine samples were collected to detect renal brush border membrane enzymes (RBBME) by ELISA and β2-microglobulin (β_2_-MG) by radioimmunoassay (RIA). The results were compared with those of 30 normal neonates. Data were statistically analyzed using SPSS13.0 software.

**Results:**

Both RBBME and β_2_-MG were found to be higher in urine in NS patients than normal controls (P < 0.01). Level of RBBME increased with the severity of NS (P **<**0.05), while urinary β_2_-MG did not (P *>*0.05). After being treated with ligustrazine, a medicine for renal function recovery, both RBBME and β_2_-MG were similarly significantly decreased comparing to untreated groups (P < 0.05). 79.7% of NS patients showed abnormal RBBME while only 52.2% had an abnormal urinary β_2_-MG (*χ*^2^=11.65，*P *< 0.01).

**Conclusion:**

RBBME was more sensitive than β_2_-MG in reflecting the renal injury in NS. Examination of RBBME effectively reflected the recovery of renal injury after treatment with ligustrazine.

## INTRODUCTION

Neonatal Scleredema (NS) is also called neonatal cold injury syndrome, which is characterized by diffuse hardening of the subcutaneous tissue, low body temperature, and edema with minimal inflammation.^1^^,^^2^ NS often affect preterm neonates in the first week of life.^3^ Serious NS may cause multiple organ dysfunctions. One of the complications of NS is impaired renal function; mainly refer to proximal tubule lesions with clinical symptoms including oliguria, anuria, proteinuria, acute tubular necrosis, and even kidney failure. Diagnosis on renal dysfunction was traditionally based on the elevated blood urea nitrogen (BUN) and creatinine (Cr), as well as decreased urine volume. However, it is difficult to achieve signs for early neonatal renal damage with these tests.

Clinically it has proven that the proximal tubule of the kidney is especially susceptible to ischemic, inflammatory or toxic events.^4^^-^^7^ The enzymes that bound to the brush border of microvillous membrane, including alkaline phosphatase (ALP), leucineaminopeptidase (LAP), γ-glutamyltransferase (γ-GT), are collectively called renal brush boarder membrane enzyme (RBBME). The shedding of the tubular epithelial membrane (and consequently the RBBME) might occur before the histopathological damage and this enzymuria could be a useful early marker of renal damage. In the present study, we evaluated clinical significance of the RBBME assay in renal injury diagnosis in NS patients. We used β2-microglobulin(β_2_-MG) as a parallel proof for the renal injury, and ligustrazine treatment as a secondary proof to observe RBBME changes while renal injury was treated.

## METHODS

***Patients: ***The study was approved by the Ethic Committee of Liaocheng People’s Hospital and the written informed consent was obtained from each patient’s parents. Patients were eligible for enrollment if NS was diagnosed and urine test showed higher RBBME above the normal range obtained from normal control (normal new born infants was enrolled as control). NS was diagnosed with typical skin harden swelling and lower body temperature. Severity of the patients was classified as shown in Table-I. Patients who needed treatment with dopamine, phentolamine, anisodamine or other vasoactive drugs were excluded from the study.

***Sample collection: ***Urine and blood samples from normal control group were obtained for one time at the clinic. Blood and urine samples from NS patients were collected at the time of hospitalization, and before and after ligustrazine treatment.

***Detection of RBBME, β2-MG, BUN and creatinine: ***Urine samples for RBBME detection were treated with preservative solution at 9:1 ratio and tested immediately or stored at -80°C freezer. RBBME were detected using the detection kit provided by Dr. Jingti Deng of Shandong University School of Medicine with ELISA method described earlier.^8^ Tests results were considered abnormal when the value was equal or higher than the mean + 2SD (standard deviation) of the normal control group. β2-MG was measured by radioimmunoassay that was routinely operated in clinical lab. Blood urea nitrogen (BUN) and creatinine (Cr) test results were also obtained from clinical labs.

***Treatment: ***Conventional treatments were applied for all patients to ensure proper management including restoration of body temperature, energy supply and fluid infusion, correction of acidosis and electrolyte imbalance, symptomatic treatment for organ malfunctioning, and if necessary, oxygen or antibiotics therapy. Patients were further randomly separated into two groups based on their enrollment number (odd number was ligustrazine group and even number was un-treated group). Ligustrazine(Shanghai Modern Hasen Pharmaceutical Co, China) was administered at 6mg/kg in 30ml 5% glucose solution *i.v.* infusion once daily for 5 consecutive days.

***Statistical analysis: ***Data were statistically analyzed using SPSS13.0 software, and expressed in mean±SD. Median was used to represent data of none normal distribution. Data comparison between groups was performed with student’s t test. Pearson correlation or Chi square test was analyzed and two-tailed probability at 0.05 was taken as significant level. The sample size of 60 patients for the study was estimated by using a two-sided t-test at the 5% significance level (= 0.05) and 80% power (= 0.2). Adjusting by 10% to account for ineligibility resulted in a final targeted sample size of 66 patients. 

## RESULTS

***General characteristics of patients: ***Sixty nine infants with NS were enrolled from June 2009 - March 2013 in our hospital, including 40 males and 29 females with an age of 8 hour - 28 day at the time of enrollment (2.8±1.2 d), birth weight ranging from 1.21 - 3.99 kg (2.68 ±0.8 kg), gestational age from 32 to 43 wks (37.8 ±2.6 wks). Based on the grading standards published in Practical Neonatology (Version 4),^9^39 cases were diagnosed as mild NS and 30 as moderate to severe (Mod/Sev)NS. Another 30 normal infants were enrolled as control, including 17 males and 13 females, 33 to 42 weeks of gestational age, birth weight 1.51 ~ 4.00Kg. Demographic data for groups of the study are listed in Table-II. No significant differences were found in sex, age, and birth weight among these groups (Table-II).

***Correlation of*** *β* ***2-MG and RBBME with the severity of NS: ***All test values from enrolled patients were showed in Table-III. Both RBBME andβ2-MG values were significantly higher in NS patients than the control group (P<0.01), while both BUN and Cr tests showed normal results. RBBME level in NS group was correlated with the severity of the disease. Significantly higher RBBME was found in Mod/Sev group than that of the mild group (p<0.05). And by linear correlation analysis, RBBME and β2-MG had a significant positive correlation (r = 0.560, p <0.01).

**Fig.1:**
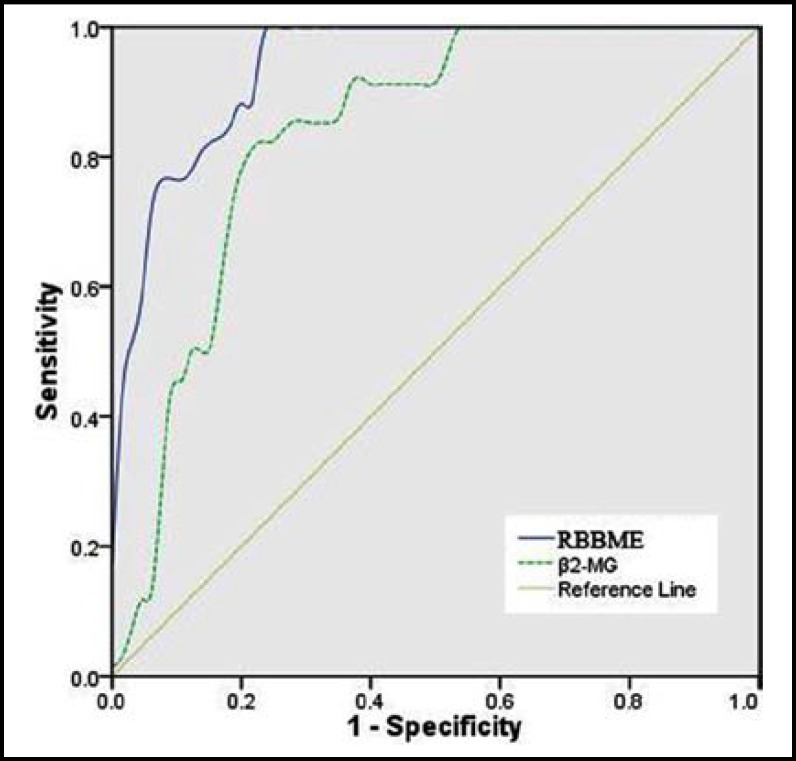
ROC curves of RBBME and β_2_-MG assay for renal injury detection. The AUCs were 0.939 and 0.834 for RBBME and β_2_-MG respectively. The solid line represents RBBME and the dotted line represents β_2_-MG. At the cutting points of 36.75 U/L for RBBME and 3.85 mg/L for β_2_-MG, both markers showed highest sensitivity

ROC curves of both RBBME and β2-MG were generated as shown in Fig.1. The area under the curve (AUC) for RBBME and β2-MG were 0.939 and 0.834 respectively, indicating higher diagnostic accuracy of RBBME for NS kidney damage. Youden index^10^ was calculated to determine the cutting points for RBBME to be 36.75U/L and β2-MG 3.85 mg/L, at which that RBBME exhibited a sensitivity of 88.2%, specificity of 81.5%, while the corresponding sensitivity of β2-MG was 82.4%, specificity 80.0% for the diagnosis of renal injury of NS.

***RBBME as an*** ***indicator for the*** ***efficacy of*** ***ligustrazine treatment: ***In order to evaluate the capability of RBBME test for reflection of renal function recovery, NS patients in each level were further divided into two groups randomly and applied ligustrazine to one group. The other group received no treatment. Ligustrazine is a Chinese herb extracts that has known function to restore normal renal function.^11^^,^^12^ Ligustrazine was administered at 6mg/kg in 30ml 5% glucose solution via iv infusion once daily for 5 consecutive days. Both RBBME and β_2_-MG decreased significantly in both mild and mod/sev groups after application of ligustrazine(p<0.01), as shown in Table-IV.

## DISCUSSION

NS is a common disease in the northern territory of China, while not many reports are seen from western countries. Its clinical manifestation is very similar to the scleremaneonatorum, whereas basic treatment method is about the same. Organ dysfunction, including renal dysfunction, is a severe complication in NS, which was found in 20% of patients (Our unpublished observation). BUN and Cr are routine clinical tests for renal function. However, these indicators were not able to reflect early renal damage, which was further proven in this study. β2-MG is a widely used indicator in clinical detection for early renal tubular dysfunction. β2-MG is filtered through the glomerulus and almost completelyre-absorbed and lysed by the proximal tubular cells.^13^ Impairment of β2-MG tubular uptake results in a raised intact β2-MG urinary excretion. Therefore, urinary β2-MG is a sensitive indicator reflecting of renal tubular dysfunction. However, multiple factors can influence the result, including β2-MG production, filtration function of glomerulus, and presence of proteinuria.^14^^-^^18^RBBME can be a more direct indicator of the tubular function that is less affected by other factors.^19^Shedding of RBBME reflects the acute tubular injury that can be detected before any other symptoms has been developed.^20^Assay of RBBME has been used in evaluation of drug-induced nephrotoxicity,^21^post transplantation kidney function surveillance,^22^ etc.

Ligustrazine, a purified and chemical identified component of a Chinese herbal remedy, has been used clinically widely in treating cardiovascular disease and improve microcirculation. It has strong effects on scavenging cytotoxic oxygen free radicals and promoting blood flow. It also has anti-platelet aggregation and radical scavenging effect.^23^ Ligustrazine has shown protective effect on early renal injury induced by various factors,^24^^-^^26^ and is able to improve microcirculation, reduce glomerular lipid peroxidation injury, delay glomerulosclerosis process, and regulate arachidonic acid metabolism, etc.^27^ Our previous study has demonstrated that ligustrazine could reduce renal dysfunction associated with attenuating lipid peroxidation (LPO), apoptosis and ICAM-1 expression.^28^ In this study ligustrazine was administered to patients with low dosages for two purposes. One was as a treatment measurement for renal injury, and second was to further verify the RBBME assay as an early marker of renal injury. A reverse of RBBME level in patients after being treated with ligustrazine would further indicate the effectiveness of the measurement. Both RBBME and β2-MG were found to be significantly declined after application of ligustrazine compared with the untreated group, suggesting that both RBBME and β2-MG were effective indicators for renal function recovery. Application of ligustrazine in this study couldn’t prove its direct effect on renal damage, but still justified the clinical the use of the medicine for NS patients. 

In this study, we found both RBBME and β_2_-MG in the mild group were significantly higher than control group (p<0.01). With the increase of severity of NS, the RBBME was found to be elevated significantly (p<0.05), whereas β_2_-MG only showed minor increase which had no statistical significance (p>0.05), suggesting that RBBME was a better indicator representing for the severity of NS than β_2_-MG. By analysis in ROC curve, RBBME exhibited to be a better marker with higher sensitivity for renal damage in NS than β_2_-MG.

Detection of RBBME has been evolved greatly to the current methodology. Deng *et al.* has developed a reliable detection methodology using specific antibodies against RBBME. The measurement is rapid, reproducible, highly specific, sensitive, and can simultaneously measuring a large number of specimens.^29^

**Table-I:** Classification of neonatal scleredema

**Type**	**Body temperature**		**Involved area**
	T _Anus_	T_Axil_ – T_Anus_*	(%, Color)
I (mild)	≥35°C	positive	<20, pale
II (moderate)	<35°C	0 or positive	20-50, dark red
III(severe)	<30°C	negative	>50, cyanotic

* T_Axil_: the axillary temperation; T_Anus_: the anal temperature;

**Table-II:** Demographic characteristics of patients

**Group**	**Number** **(M/F)**	**Age** **(median, day)**	**Gestational Term (mean** **±SD**, **wks)**	**Weight** **(mean** **±SD, ** **kg)**
Mild	39 (22/17)	3.6	38.1±2.5	2.91±0.72
Mod/Sev	30 (18/12)	2.4	35.8±2.7	2.79±0.69
Control	30 (17/13)	3.8	37.5±2.4	2.53±0.83

**Table-III:** Comparison of RBBME and β2-MG between Mild and Mod/Sev groups

**Group**	**Number**	**RBBME** **(U/L)**	**β**_2_ **-MG ** **(mg/L)**	**BUN** **(mmol/L)**	**Cr** **(µmol/L)**
Mild	39	38.57±6.70^a^	4.45±1.18^a^	5.40±1.80	72.48±19.03
Mod/Sev	30	42.06±7.59^a^,^b^	4.91±1.49 a, c	6.30±1.78	80.77±19.13
Control	30	23.19±5.62	2.49±0.77	5.05±1.14	69.00±11.97

a *P*<0.01when compared with control group;

b *P*< 0.05,

c *P>*0.05 when compared with mild group.

**Table-IV:** Comparison of RBBME and β2-MG as indicators for efficacy of ligustrazine treatment

**Group**		**Number**	**RBBME (U/L)**	**β**_2_ **-MG (mg/L)**
Mild	Treated	24	26.86±6.00*	2.90±0.90*
	Untreated	15	31.11±5.72	3.47±0.82
Mod/Sev	Treated	11	29.80±6.58*	3.19±0.69*
	Untreated	19	35.20±6.33	3.70±0.66

* *P*< 0.05 by comparing with the corresponding untreated group.

Theologically the shedding of RBBME from brush border of microvillous membrane would be an early sign of renal function defect. We have found that both RBBME and β_2_-MG exhibited positive signs for renal damage while BUN and Cr were normal. This suggests that RBBME can be used as early detection of renal damage in NS patients. Since the value of RBBME has a positive correlation with the severity of NS, we propose that RBBME may be a more effective indicator for renal damage than β_2_-MG in NS patients.

In summary, detection of urine RBBME was useful indicator for renal dysfunction as well as treatment efficacy. With the improvement of the methodology, RBBME assay is likely to replace β_2_-MG for early detection and better accuracy.
